# A subset of ocular adnexal marginal zone lymphomas may arise in association with IgG4-related disease

**DOI:** 10.1038/srep13539

**Published:** 2015-08-27

**Authors:** Kyotaro Ohno, Yasuharu Sato, Koh-ichi Ohshima, Katsuyoshi Takata, Tomoko Miyata-Takata, Mai Takeuchi, Yuka Gion, Tomoyasu Tachibana, Yorihisa Orita, Toshihiro Ito, Steven H. Swerdlow, Tadashi Yoshino

**Affiliations:** 1 Department of Pathology, Okayama University Graduate School of Medicine, Dentistry, and Pharmaceutical Sciences, Okayama, Japan; 2 Division of Pathophysiology, Okayama University Graduate School of Health Sciences, Okayama, Japan; 3 Department of Ophthalmology, National Hospital Organization Okayama Medical Center, Okayama, Japan; 4 Department of Otolaryngology, Himeji Red Cross Hospital, Himeji, Japan; 5 Department of Otolaryngology, Head and Neck Surgery, Okayama University Graduate School of Medicine, Dentistry, and Pharmaceutical Sciences, Okayama, Japan; 6 Department of Immunology, Nara Medical University, Nara, Japan; 7 Department of Pathology, Division of Hematopathology, University of Pittsburgh School of Medicine, Pittsburgh, PA, USA

## Abstract

We previously suggested a relationship between ocular immunoglobulin (Ig)G4-related disease (IgG4-RD) and marginal zone lymphomas (MZLs). However, the cytokine background associated with these disorders and whether it differs between ocular adnexal MZLs with (IgG4-associated MZL) and without (IgG4-negative MZL) numerous IgG4^+^ plasma cells are unknown. In this study, we identified the mRNA expression pattern of Th2 and regulatory T-cell (Treg) cytokines in IgG4-RD and in IgG4-associated MZL and IgG4-negative MZL using real-time polymerase chain reaction analysis. Ocular IgG4-RD and IgG4-associated MZL exhibited significantly higher expression ratios of interleukin (IL)-4/β-actin, IL-10/β-actin, IL-13/β-actin, transforming growth factor (TGF) β1/β-actin, and FOXP3/β-actin than did IgG4-negative MZL (p < 0.05). This finding further supports our prior observations that a significant subset of ocular MZLs arises in the setting of IgG4-RD. Furthermore, the presence of a different inflammatory background in IgG4-negative MZLs suggests that IgG4-associated MZLs may have a different pathogenesis.

Immunoglobulin (Ig)G4-related disease (IgG4-RD) is a systemic syndrome characterized by dense lymphoplasmacytic infiltrates that are rich in IgG4^+^ plasma cells, fibrosis in the involved organ, and elevated serum IgG4 levels. IgG4-RD can affect almost any organ, including the pancreas, hepatobiliary duct, lacrimal and salivary glands, lung, kidney, retroperitoneum, aorta, and lymph nodes^1^,^2^,^3^,^4^,^5^,^6^,^7^,^8^,^9^,^10^,^11^.

Recent reports have described upregulation of T-helper-2 cells (Th2) and regulatory T-cell (Treg) cytokines in tissues with IgG4-RD, suggesting that the immune reaction mediated by these cytokines is responsible for the lesions^12^,^13^. This is in contrast to most extranodal marginal zone lymphomas (MZLs) that have a Th1 type inflammatory background, but similar to the Th2 background seen in a large cutaneous MZL subset that is also often IgG4-positive^14^,^15^,^16^.

Previously, we reported cases of ocular adnexal MZLs with numerous IgG4^+^ plasma cells that fulfilled the histological diagnostic criteria for IgG4-RD; therefore, we suggested that MZLs can arise in a background of IgG4-RD^1^. However, the expression pattern of cytokines in MZL lesions with IgG4^+^ plasma cells has not been clarified.

In this study of ocular IgG4-RD and MZLs with (IgG4-associated MZL) and without (IgG4-negative MZL) numerous IgG4^+^ plasma cells, we aimed to identify the mRNA expression patterns of Th2 and Treg cytokines and to determine the inflammatory background associated with benign and neoplastic ocular lymphoplasmacytic proliferations with numerous IgG4^+^ plasma cells that is distinct from that associated with ocular IgG4-negative MZL.

## Material and Methods

### Samples and clinical review

Formalin-fixed excisional biopsies from the ocular adnexal region of patients were selected, including 11 patients with IgG4-RD, 11 with IgG4-negative MZL, and 6 with IgG4-associated MZL (Table 1). All MZL lesions were primary tumors, and there was no other organ involvement. None of the patients were treated prior to the biopsy. Clinical data including serum IgG4 and IgG levels were obtained when available. The IgG4 and IgG levels were measured by routine laboratory blood tests. Informed consent for the use of their samples in research was obtained from all patients.

### Real-time quantitative polymerase chain reaction (PCR)

The following evaluations were carried out in accordance with the approved guidelines. All experimental protocols were approved by the Institutional Review Board at Okayama University. Total RNA was extracted from the paraffin-embedded sections of all samples using an miRNeasy FFPE Kit (QIAGEN, Valencia, CA, USA). cDNA was prepared using a SuperScript VILO MasterMix kit (Invitrogen, Carlsbad, CA, USA). Multiplex real-time PCR was performed for quantitative analysis, according to a standard protocol, using TaqMan Gene Expression Assays (Applied Biosystems, Foster City, CA, USA), a Step One Plus Real-Time PCR System (Applied Biosystems), and specific primers and probes for FOXP3 (Hs01085834_m1), transforming growth factor (TGF)β1 (Hs00998133_m1), interleukin (IL)-4 (Hs00174122_m1), IL-5 (Hs00174200_m1), IL-10 (Hs00961622_m1), IL-13 (Hs00174379_m1), and β-actin (Hs99999903_m1) (Applied Biosystems). The PCR cycling conditions were as follows: 20 s at 95 °C, 50 cycles of 1 s at 95 °C, and 20 s at 60 °C. The expression of each target was normalized to that of β-actin, which was used as an endogenous control.

### Histological examination, immunohistochemistry, and *in situ* hybridization

Serial sections (4 μm) were cut from the block of paraffin-embedded tissue, stained with hematoxylin and eosin, and used for the following immunohistochemical stains: CD20 (L26 [1:400]; DAKO, Glostrup, Denmark), CD3 (LN10 [1:200]; Novocastra, Newcastle, UK), CD5 (4c7 [1:50]; Novocastra), CD10 (56C6 [100:1]; Novocastra), CyclinD1 (SP4 [1:50]; Nichirei, Tokyo, Japan), Ki-67 (MIB-1 [1:2500]; Novocastra), IgG (polyclonal [1:20,000]; DAKO), and IgG4 (HP6025 [1:10000]; The Binding Site, Birmingham, UK). Following immunostaining using an automated Bond Max stainer (Leica Biosystems, Melbourne, Germany), the numbers of IgG4^+^ and IgG^+^ cells were estimated in areas with the highest density of IgG4^+^ cells. In accordance with the consensus statement on the pathological features of IgG4-RD^17^, three different high-power fields (HPFs) (total magnification, ×400) were examined to calculate the average number of IgG4^+^ cells per HPF and the IgG4^+^/IgG^+^ cell ratio. *In situ* hybridization was also performed for κ and λ-light chains (Leica Biosystems) using a Bond Max stainer.

### Molecular genetic analysis

PCR molecular genetic analysis for immunoglobulin heavy chain gene rearrangements was performed as previously described^18^,^19^,^20^. The primers used in this study were: 5′-TGG[A/G]TCCG[C/A]CAG[G/C]C[T/C][T/C]C[A/C/G/T]GG-3′ as an upstream consensus V-region primer; 5′-TGAGGAGACGGTGACC-3′ as a consensus J-region primer, and 5′-GTGACCAGGGT[A/C/G/T]CCTTGGCCCCAG-3′ as a consensus J-region primer^18^,^19^,^20^.

### Statistical analysis

All statistical analyses were performed using the Mann–Whitney *U*-test with SPSS software (version 14.0; SPSS, Chicago, IL, USA).

## Results

### Histological/phenotypic examinations

#### IgG4-related disease

**Figure 1:**
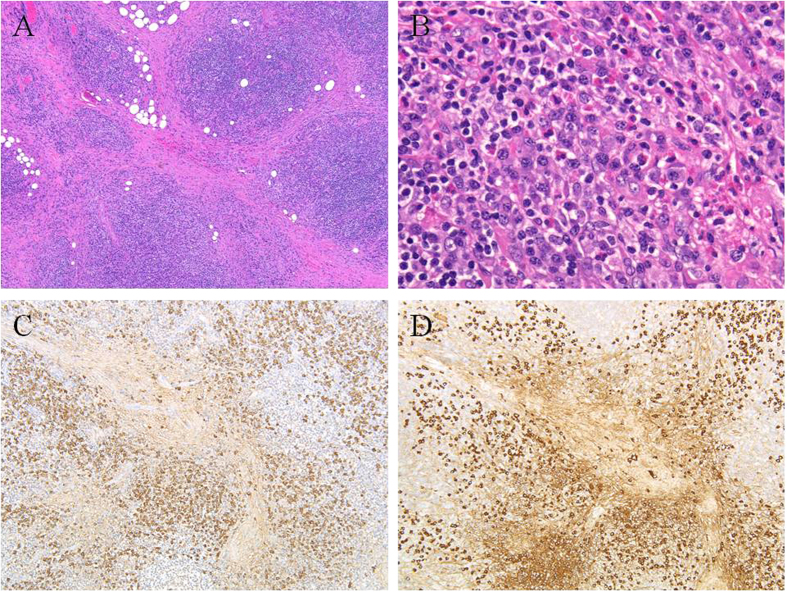
IgG4-related disease (case 6). Marked lymphoplasmacytic infiltration with dense fibrosis, scattered eosinophils, and interspersed reactive lymphoid follicles are shown (**A,B**) (hematoxylin & eosin). Numerous IgG^+^ (**C**) and IgG4^+^ (**D**) cells are present with an IgG4^+^/IgG^+^ cell ratio >40%.

Biopsies from the 11 patients with IgG4-RD showed marked lymphoplasmacytic infiltration with or without dense fibrosis, scattered eosinophils, and interspersed reactive lymphoid follicles. No light-chain restrictions were noted. All cases demonstrated >100 IgG4^+^ cells/HPF, and the IgG4^+^/IgG^+^ cell ratio was >40% (Fig. 1).

#### IgG4-negative marginal zone lymphoma

**Figure 2:**
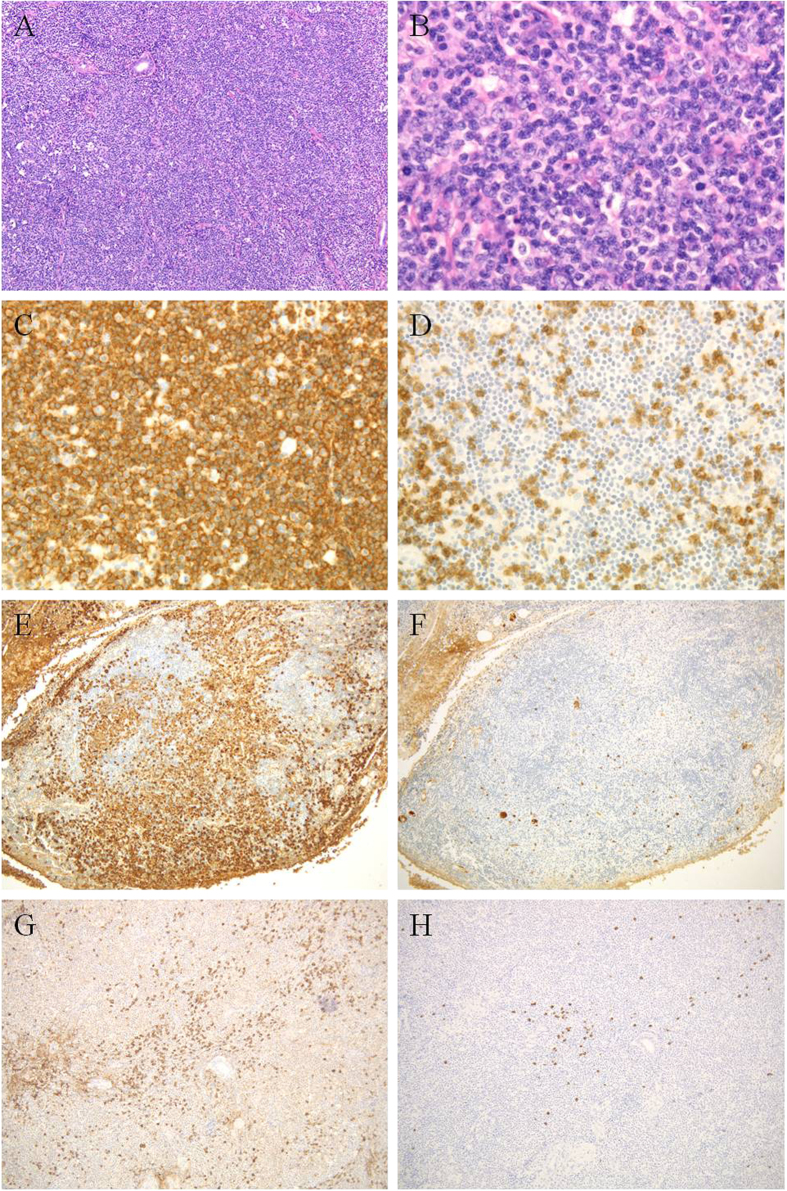
IgG4-negative marginal zone lymphoma (case 16). There is a diffuse proliferation of small- to medium-sized lymphoid cells (**A**,**B**) that are CD20^+^ (**C**) and CD3^−^ (**D**). Numerous Igκ^+^ plasma cells are present (**E**), but only very few Igλ^+^ plasma cells (**F**). Some IgG^+^ (**G**) and IgG4^+^ (**H**) cells are present but the IgG4^+^/IgG^+^ cell ratio is <40%.

The 11 IgG4-negative MZLs showed dense infiltration by small- to medium-sized CD20^+^, CD3^−^, CD5^−^, and CD10^−^ lymphoid cells with a Ki-67 labeling index <10% (Fig. 2). Fibrosis and eosinophil infiltration were rarely present. In 3 specimens (cases 15, 16, and 18), *in situ* hybridization was performed, and polytypic plasma cells were detected. PCR revealed clonal immunoglobulin heavy chain rearrangement in 1 of 2 cases tested (case 22 but not case 15). All cases revealed few, if any, IgG4^+^ cells, and the IgG4^+^/IgG^+^ cell ratio was <40%.

#### IgG4-associated marginal zone lymphoma

**Figure 3:**
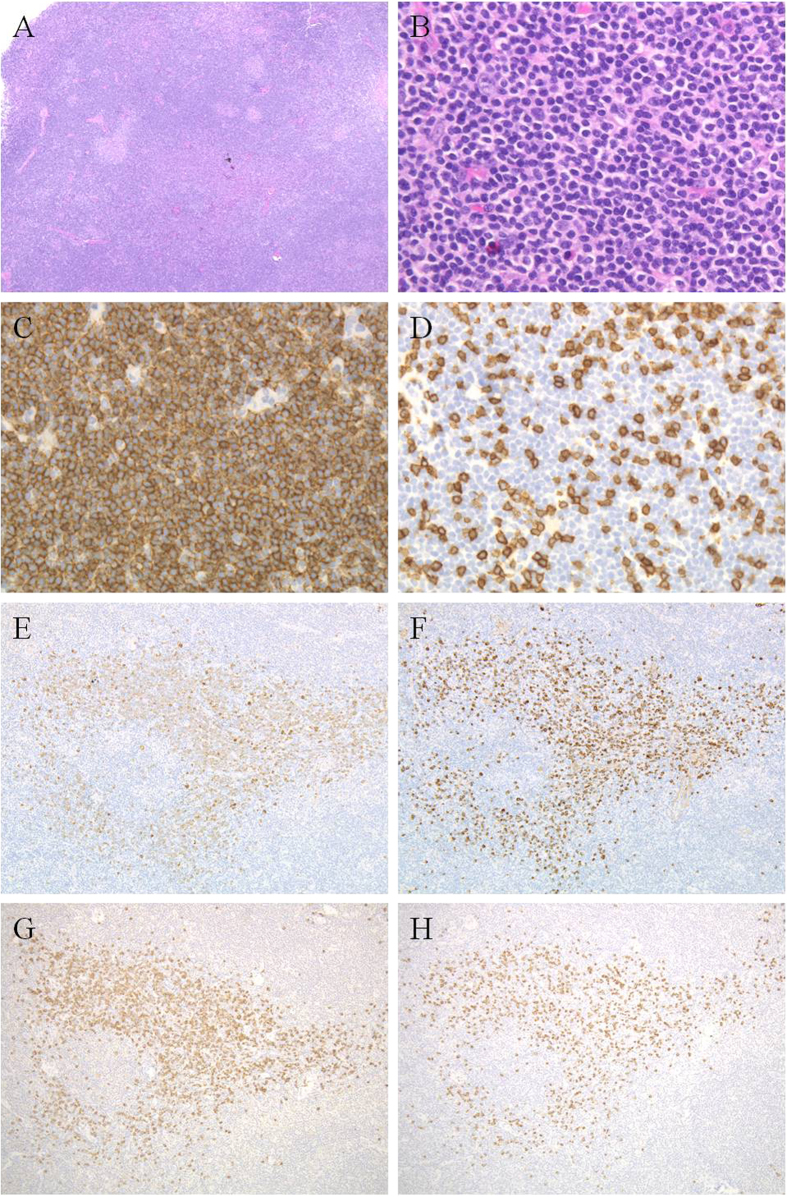
IgG4-associated marginal zone lymphoma. The diffusely proliferating small- to medium-sized lymphoid cells [(**A**,**B**) case 26] are CD20^+^ (**C**) and CD3^−^ (**D**). Infiltration of many IgG^+^ (**E**) and IgG4^+^ (**F**) cells are observed, but these cells are polytypic (**G**) (Igκ) and (**H**) (Igλ). The IgG4^+^/IgG^+^ cell ratio is >40%.

**Figure 4:**
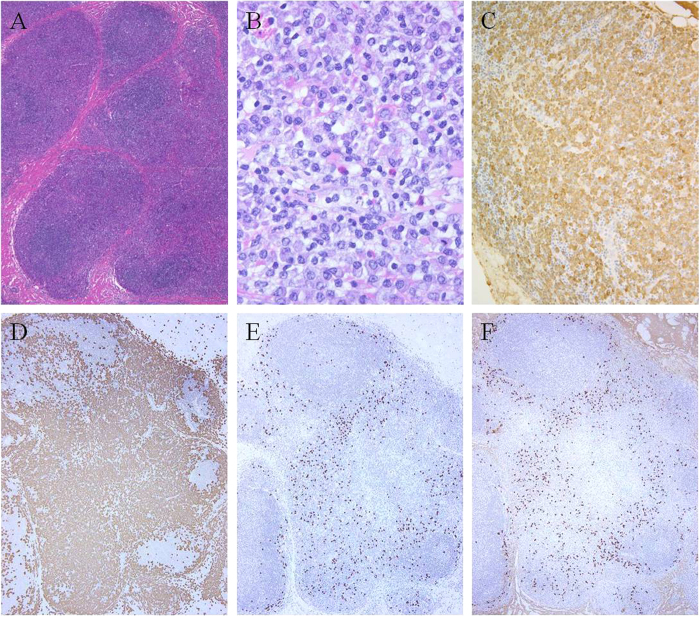
IgG4-associated marginal zone lymphoma (case 28). Numerous plasmacytic cells are present (**A**,**B**) that are IgG^+^ (**C**). The lymphoma cells are κ light chain-restricted. (**D**) Igκ-*in situ* hybridization and (**E**) Igλ-*in situ* hybridization. Admixed IgG4^+^ plasma cells that appear to be polytypic are present (**F**).

The 6 IgG4-associated MZLs were histologically and phenotypically similar to the IgG4-negative MZLs except that there were numerous IgG4^+^ cells, and fibrosis was present in 3 cases (cases 26, 27, and 28) (Table 1, Fig. 3). All cases had either clonal immunoglobulin heavy chain rearrangement (cases 23, 25, and 26) and/or were monotypic based on *in situ* hybridization for κ and λ (cases 24, 25, 27, and 28). Based on the distribution of the IgG4^+^ cells and non-dominant light chain staining, the IgG4^+^ cells appeared to be polytypic. In all 6 cases, >100 IgG4^+^ cells/HPF were detected; therefore, these cases fulfilled the histological diagnostic criteria for IgG4-RD. Furthermore, in 5 cases, the IgG4^+^/IgG^+^ cell ratio was >40%. In case 28, the IgG4^+^/IgG^+^ cell ratio was not estimated because the lymphoma cells present were IgG positive, and the much fewer IgG4^+^ cells appeared to reflect a polytypic IgG^+^ population that could not be quantitated (Fig. 4).

### Serological analysis

Serum IgG4 and IgG levels were determined in 7 patients with ocular IgG4-RD, in 2 patients with IgG4-negative MZL, and in 4 patients with IgG4-associated MZL (Table 1). Serum IgG4 levels were >135 mg/dL (reference range, 4.8–105) in 5 of 7 tested patients with IgG4-RD, 1 of 2 patients with IgG4-negative MZL, and 3 of 4 patients with IgG4-associated MZL.

### Expression pattern of Th2 and Treg cytokines

**Figure 5:**
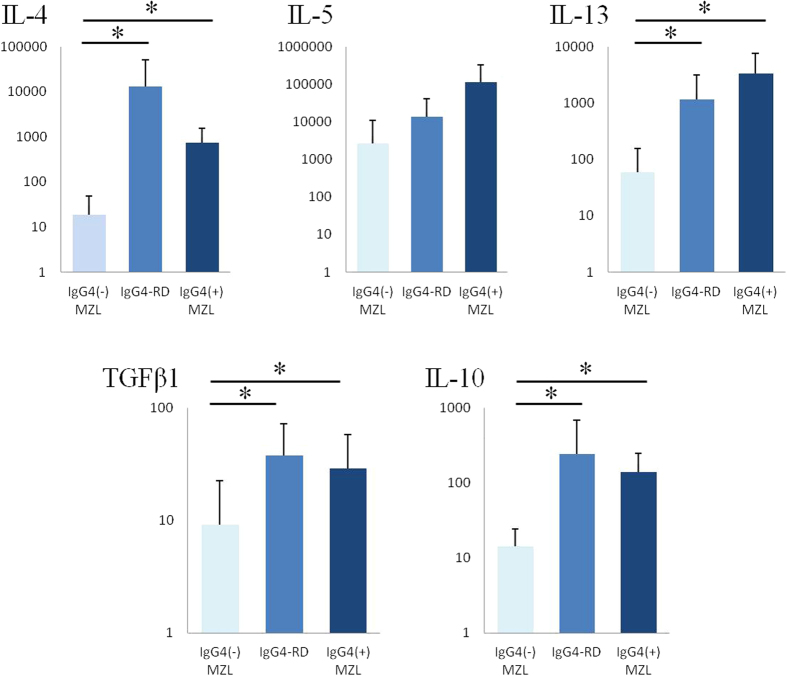
Cytokine levels were measured by relative quantification of mRNA. mRNA expression of Th2 cytokines (IL-4, IL-13) and regulatory cytokines (TGFβ1, IL-10) are significantly higher in the ocular adnexal regions from biopsies with IgG4-related disease and IgG4-associated marginal zone lymphoma than in the ocular adnexal regions from IgG4-negative marginal zone lymphomas. The expression levels of IL-5 are not significantly different. Data represent mean ± standard deviation (SD) values. Significant differences between groups were determined using the Mann-Whitney U test. (*p < 0.05). IgG4^−^ MZL, IgG4-negative marginal zone lymphoma; IgG4-RD, IgG4-related disease; IgG4^+^ MZL, IgG4-associated marginal zone lymphoma.

The expressions of IL-4, IL-5, IL-10, IL-13, TGFβ1, and β-actin in the samples were quantitatively analyzed using real-time PCR. Ocular IgG4-RD and the IgG4-associated MZL samples exhibited significantly higher expression ratios of IL-4/β-actin, IL-10/β-actin, IL-13/β-actin, and TGFβ1/β-actin than did the IgG4-negative MZL samples (p < 0.05) (Fig. 5). No significant differences were found for IL-5/β-actin expression.

### Expression of FOXP3

**Figure 6:**
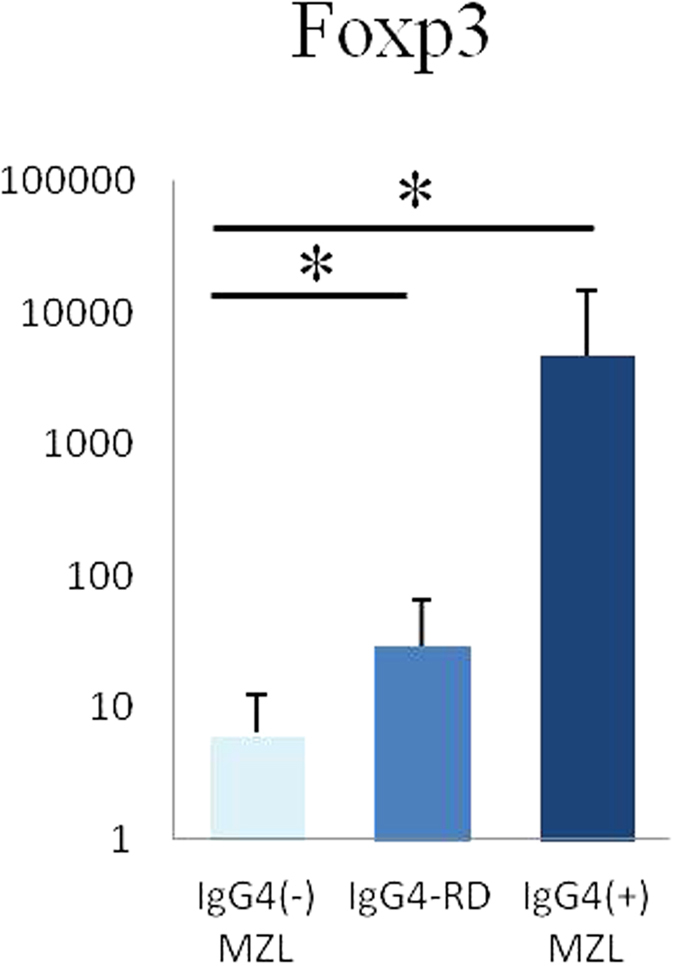
FOXP3 expression levels were measured by relative quantification of mRNA. mRNA expressions of FOXP3 are significantly higher in the ocular adnexal regions from biopsies with IgG4-related disease and IgG4-associated marginal zone lymphoma than in the ocular adnexal resions from IgG4-negative marginal zone lymphomas. (*p < 0.05). IgG4^−^ MZL, IgG4-negative marginal zone lymphoma; IgG4-RD, IgG4-related disease; IgG4^+^ MZL, IgG4-associated marginal zone lymphoma.

The ocular IgG4-RD and IgG4-associated MZL biopsies exhibited significantly higher expression ratios of FOXP3/β-actin than did the IgG4-negative MZL specimens (p < 0.05) (Fig. 6).

## Discussion

The Th1/Th2 balance is considered important for healthy immune responses, and a Th1/Th2 imbalance can cause immune-mediated disease. Th1-dominant immune responses are noted in patients with rheumatoid arthritis, type 1 diabetes, and multiple sclerosis, whereas Th2-dominant immune responses are observed in patients with type 1 hypersensitivity disorders, such as allergies or asthma^21^. Extranodal MZLs are considered to arise in a background of chronic inflammation. High levels of Th1-type cytokines (interferon [IFN]-γ and IL-2) are typically found in the tumor environment of many mucosa associated lymphoid tissue (MALT) lymphomas^22^,^23^. Moreover, most extranodal MZLs express high levels of CXCR3, which is the receptor for IFN-γ-induced chemokines^24^,^25^,^26^. In contrast, a major subset of cutaneous MZLs typically shows heavy chain class switching and has a Th2 background^14^,^15^. In addition, 39% of cutaneous MZLs with plasmacytic differentiation are reported to be IgG4^+^, whereas an ocular MZL was the only non-cutaneous IgG4^+^ MZL found in the same study^16^.

Recently, high levels of Th2 cytokines (IL-4 and IL-13) and Treg cytokines (TGFβ1 and IL-10) have been detected in tissues with IgG4-RD^12^. Costimulation with IL-4 and IL-10 is suggested to cause class switching to IgG4^27^. Furthermore, upregulation of IL-13 induces eosinophil infiltration, and TGFβ1 is considered to cause fibrosis in IgG4-RD lesions. In this study, we found higher levels of Th2 and Treg cytokines (including FOXP3) in lesions from patients with either IgG4-RD or ocular IgG4-associated MZL than in samples from patients with ocular adnexal IgG4-negative MZL. However, the expression levels of IL-5, which is a Th2 cytokine that promotes eosinophil infiltration, were not significantly different between sample types. These results indicate that IgG4-associated MZL is characterized by the upregulation of Th2 and Treg cytokines, similar to IgG4-RD. Despite sharing a similar inflammatory background with class-switched cutaneous MZLs, and very much unlike most other MALT lymphomas, the ocular cases, with one exception, had IgG4^+^ plasma cells that were polytypic and not part of the neoplasm. An explanation for this difference remains to be established, and the heavy chain expressed by the ocular lymphomas is uncertain.

The mechanism for the development of ocular adnexal MZL is not clear. However, recent reports indicated that *Chlamydia psittaci* infection is associated with the development of ocular adnexal MZL^28^,^29^,^30^. We previously suggested that ocular adnexal MZLs may arise in the setting of ocular IgG4-RD^1^, and others have reported cases of ocular adnexal lymphoma with IgG4^+^ cells, suggesting that they arose from IgG4-RD^31^. Moreover, a case involving two types of mass lesions in the same ophthalmic region has also been reported^32^. One of the lesions was typical of IgG4-RD, and the other was an MZL admixed with IgG4^+^ plasma cells. Because the two lesions were adjacent to each other, the MZL may have arisen in the setting of the IgG4-RD lesion. These reports and the results of the present study suggest that a subset of ocular MZLs may arise in the setting of IgG4-RD.

Patients with IgG4-RD are considered to be at high risk of MZLs as well as other malignancies, with malignancy considered a possible complication of IgG4-RD. Lung and colon cancers, as well as malignant lymphomas, are reported to occur in 10.4% of patients with IgG4-RD, an incidence that is approximately 3.5 times higher than that in the general population^33^. Although the mechanism through which neoplasms arise in a background of IgG4-RD remains unknown, a recent report suggests that IgG4 subclass antibodies impair antitumor immunity against melanomas^34^. The authors reported that IgG4 antibodies did not induce antitumor immunity, unlike IgG1 antibodies; rather, they inhibited the antitumor activity of the IgG1 antibodies. Therefore, although IgG4 subclass antibodies do not specifically induce neoplasms, they may create an environment where induced malignant neoplasms are more likely to grow.

In the current study, upregulation of FOXP3 was detected in cases of IgG4-RD and IgG4-associated MZL lesions. This is consistent with an earlier report describing the presence of numerous FOXP3^+^ cells in IgG4-RD^12^. FOXP3 is a marker of Tregs and is a central control element in their development and function. Because Tregs have been reported to suppress inflammation, including tumor immunity^35^, FOXP3^+^ Tregs may infiltrate lesions associated with IgG4-RD and further suppress tumor immunity, also promoting the growth of neoplasms.

Although steroid therapy is effective in IgG4-RD, the most effective treatment for MZLs arising in the setting of IgG4-RD is uncertain. Although evaluation of additional cases of IgG4-associated MZLs is needed to determine the treatment and prognosis, radiation or chemotherapy may be necessary. Therefore, when IgG4-RD is diagnosed, the lesion should be assessed to determine if it includes an MZL component. The possible tumor-promoting environment of IgG4-RD supports treatment for these patients before an MZL arises, in addition to ongoing follow-up.

## Conclusion

IgG4-associated MZL is characterized by the upregulation of Th2 and regulatory cytokines, as is the case in IgG4-RD, but unlike the cytokine background of IgG4-negative MZLs. The current results further suggest that a subset of MZLs may arise in an IgG4-RD setting and that this subset of MZLs may have a different pathogenesis than IgG4-negative MZLs.

## Additional Information

**How to cite this article**: Ohno, K. *et al.* A subset of ocular adnexal marginal zone lymphomas may arise in association with IgG4-related disease. *Sci. Rep.* **5**, 13539; doi: 10.1038/srep13539 (2015).

## Figures and Tables

**Table 1:** Histological and serological findings.

Case No.	Diagnosis	Age	Sex	Anatomical location	IgG4^+^ cells (/HPF)	IgG^+^ cells (/HPF)	IgG4^+^/IgG^+^ cell ratio	Serum IgG4 (mg/dL) [4.8–105]	Serum IgG (mg/dL) [870–1,700]	Serum IgG4/IgG ratio
1	IgG4-RD	47	F	lacrimal gland	113	135	0.837	n.e.	n.e.	
2	IgG4-RD	60	M	orbit	178	309	0.575	n.e.	n.e.	
3	IgG4-RD	66	F	lacrimal gland	260	365	0.713	106	1400	0.076
4	IgG4-RD	73	M	lacrimal gland	137	198	0.690	136	1437	0.095
5	IgG4-RD	56	F	lacrimal gland	177	275	0.642	n.e.	n.e.	
6	IgG4-RD	61	M	orbit	298	440	0.678	3320	4168	0.797
7	IgG4-RD	66	M	lacrimal gland	365	409	0.892	969	2454	0.395
8	IgG4-RD	60	F	orbit	184	223	0.825	120	1499	0.080
9	IgG4-RD	58	F	orbit	207	248	0.836	376	1351	0.278
10	IgG4-RD	36	F	orbit	160	174	0.918	561	1958	0.287
11	IgG4-RD	72	M	orbit	311	474	0.656	n.e.	n.e.	
12	IgG4^−^ MZL	74	M	conjunctiva	0	9	0.000	n.e.	n.e.	
13	IgG4^−^ MZL	56	F	conjunctiva	16	108	0.146	n.e.	n.e.	
14	IgG4^−^ MZL	69	M	lacrimal gland	44	259	0.171	n.e.	n.e.	
15	IgG4^−^ MZL	72	M	conjunctiva	49	164	0.297	n.e.	n.e.	
16	IgG4^−^ MZL	69	M	orbit	47	669	0.071	n.e.	n.e.	
17	IgG4^−^ MZL	66	F	lacrimal gland	0	15	0.000	19.3	962	0.020
18	IgG4^−^ MZL	71	F	lacrimal gland	25	605	0.041	n.e.	n.e.	
19	IgG4^−^ MZL	61	M	orbit	0	4	0.000	n.e.	n.e.	
20	IgG4^−^ MZL	82	M	orbit	0	26	0.000	166	1520	0.109
21	IgG4^−^ MZL	39	M	conjunctiva	2	17	0.100	n.e.	n.e.	
22	IgG4^−^ MZL	74	F	conjunctiva	1	12	0.111	n.e.	n.e.	
23	IgG4^+^ MZL	57	F	lacrimal gland	128	183	0.699	n.e.	n.e.	
24	IgG4^+^ MZL	72	M	orbit	128	152	0.838	760	2709	0.281
25	IgG4^+^ MZL	65	F	lacrimal gland	143	300	0.475	116	1450	0.080
26	IgG4^+^ MZL	56	M	orbit	189	219	0.863	475	1536	0.309
27	IgG4^+^ MZL	42	F	lacrimal gland	123	152	0.807	n.e.	n.e.	
28	IgG4^+^ MZL	71	F	lacrimal gland	147			450	1844	0.244

IgG4-RD, IgG4-related disease; IgG4^−^ MZL, IgG4-negative marginal zone lymphoma; IgG4^+^ MZL, IgG4-associated marginal zone lymphoma; n.e., not examined. Normal ranges of serum IgG and IgG4 are shown in square brackets. In case no. 28, number of IgG^+^ cells is not shown because tumor cells produce IgG.
